# *Giardia duodenalis* in Wildlife: Exploring Genotype Diversity in Italy and across Europe

**DOI:** 10.3390/pathogens11010105

**Published:** 2022-01-16

**Authors:** Isabel Guadano Procesi, Margherita Montalbano Di Filippo, Claudio De Liberato, Andrea Lombardo, Giuseppina Brocherel, Stefania Perrucci, David Di Cave, Federica Berrilli

**Affiliations:** 1 Department of Clinical Sciences and Translational Medicine, Parasitology, Faculty of Medicine, University of Rome “Tor Vergata”, Via Montpellier 1, 00133 Rome, Italy; isabel.guadano.procesi@uniroma2.it (I.G.P.); dicave@uniroma2.it (D.D.C.); 2 PhD Program in Evolutionary Biology and Ecology, Department of Biology, University of Rome “Tor Vergata”, 00133 Rome, Italy; 3 Istituto Superiore di Sanità, Viale Regina Elena 299, 00161 Rome, Italy; 4 Istituto Zooprofilattico Sperimentale del Lazio e della Toscana “M. Aleandri”, Via Appia Nuova, 1411, 00178 Rome, Italy; claudio.deliberato@izslt.it (C.D.L.); andrea.lombardo@izslt.it (A.L.); giuseppina.brocherel@izslt.it (G.B.); 5 Dipartimento di Scienze Veterinarie, Università di Pisa, Viale delle Piagge 2, 56124 Pisa, Italy; stefania.perrucci@unipi.it

**Keywords:** *Giardia duodenalis*, wildlife, multi-locus genotyping (MLG), assemblages, sub-assemblages, Italy, Europe

## Abstract

Fragmented data are so far available on genotype diversity of *G. duodenalis* in wildlife in different countries in Europe, in particular, in Italy. In the present study, *G. duodenalis* sequences obtained from different Italian wild animals [12 porcupines (*Hystrix cristata*), 4 wild boars (*Sus scrofa*), 1 wolf (*Canis lupus italicus*), 6 Alpine chamois (*Rupicapra rupicapra rupicapra*)] were compared with those available from wild host species in Europe to add new data on the geographic distribution of *Giardia* assemblages/sub-assemblages and their transmission patterns among natural hosts. Thirty-eight sequences were obtained by MLG analysis (*SSU-rRNA*, *bg*, *gdh*, and *tpi* genes) and subsequently compared by phylogenetic and network analyses with those from wild species monitored in the last decades in Europe. The results revealed the presence of potentially zoonotic (A-AI, A-AII from wild boar; B from porcupine) and host-adapted (D from wolf; E, A-AIII from chamois) assemblages and sub-assemblages and represent the first report for Italian wild boar. The analysis did not find any evidence of spatial or host segregation for specific genetic variants, mostly shared between different hosts from different European countries. However, conflicting evidence was found in genotypic assignment, advocating for data improvement and new genomic approaches.

## 1. Introduction

Parasites in wildlife are a significant component of biodiversity, and their life cycles depend on the ecological networks in which they live [1,2,3], representing fundamental elements of healthy ecosystems [4,5]. However, many parasites can be a risk for threatened species because of their impacts on host populations, occasionally causing severe population declines and thus affecting conservation efforts [6,7]. In the last decades, considerable advances have been achieved in the knowledge of parasites in wildlife and on their complex interactions with the hosts, but comprehensive pictures of taxa distribution and prevalence within specific epidemiological contexts are frequently missing.

*Giardia* Kunstler, 1882 is one of the most common intestinal protozoa, transmitted through fecal deposition of cysts by an infected host. Transmission of this parasite via contaminated water and/or food to other hosts, such as domestic animals or humans, may therefore be favored by wild animals that spread cysts in the environment. To date, eight species have been described within this genus: *Giardia agilis*, *Giardia ardeae*, *Giardia psittaci*, *Giardia microti*, *Giardia muris*, *Giardia duodenalis*, and recently, *Giardia paramelis* and *Giardia cricetidarum* [8], varying in host preferences. *Giardia duodenalis* (also known as *G. lamblia* and *G. intestinalis*) is the only species parasitizing humans as well as many domestic and wild animals. This parasite exhibits considerable genetic diversity, with eight assemblages (A–H) recognized mostly by a multilocus genotyping approach (MLG), relying on different genetic markers [9,10]. Assemblages A and B can infect humans and animals, and different sub-assemblages (AI-AII-AIII and BIII-BIV) were identified within the two groups. Sub assemblages AI and AIII are found primarily in animals, while AII is largely detected in humans [8]. Conversely, due to the higher sequence diversity but lower phylogenetic resolution at standard loci, more complex appears the molecular characterization of assemblage B in sub-assemblages, likely composed of several genetically distinct lineages or clonal complexes [11]. Assemblages C–H show high specificity to different animal hosts [9]. Wild animals can be infected with different *G. duodenalis* assemblages/sub-assemblages, thus making it difficult to determine the role of wildlife, including invasive species, in the epidemiology of *Giardia* infection [9,12].

Despite the importance of this parasite in the broader ecological context, scanty and fragmented data are so far available on the genotype diversity of *G. duodenalis* in wildlife from different countries in Europe [13,14,15,16,17] and, more specifically, from Italy [18,19,20,21].

In the present work, *G. duodenalis* isolates from wild mammals in Italy were characterized by MLG analysis of the *SSU-rRNA, bg*, *gdh*, and *tpi* genes and compared with sequences previously obtained from wild species monitored in the last decades in Europe, with the aims of: (i) adding new data on the genotype of *G. duodenalis* from wildlife in Italy, (ii) providing a more comprehensive picture of the geographical distribution of *G. duodenalis* genetic variants in wildlife within the European scenario.

## 2. Results

In the present study, 38 good quality sequences at the 4 loci analyzed were achieved from 23 *G. duodenalis* isolates obtained from Alpine chamois (*Rupicapra rupicapra rupicapra*), wild boar (*Sus scrofa*), wolf (*Canis lupus italicus*), and porcupine (*Hystrix cristata*), as detailed in Table 1.

### 2.1. Genotyping of the Italian Samples at the SSU-rRNA Locus

Sequence analysis of the SSU-rRNA gene fragment allowed to assign all the 23 isolates to their respective *G. duodenalis* assemblages (see Table 1). As expected for this locus, no differences among the sequences here analyzed were observed within each assemblage. Seven sequences [four wild boar (SS1-SS4) and three Alpine chamois (RR7, RR8, RR12)] were identified as assemblage A, showing 100% identity (174/174; 100% query coverage) to *Giardia* isolates from a variety of hosts and localities [e.g., cat in Denmark (GenBank accession number: MN263894), human in Colombia (GenBank accession number: MG924430), a dog from Malaysia (GenBank accession number: KJ027399)]. Assemblage B was represented by 12 sequences identified in all isolates from porcupines (HC1–HC12), showing 100% identity (175/175; 100% query coverage) with many sequences worldwide (e.g., GenBank accession number: MG924431 from a human in Colombia and GenBank accession number: LC341260 from a cat in Japan).

Comparison of the nucleotide sequence from the wolf (CL1) revealed 100% of identity (175/175; 100% query coverage) to *Giardia* assemblage D from a dog in Australia (AF199443), whereas the sequences from two Alpine chamois (RR6, RR9) showed to be identical (174/174; 100% query coverage) with assemblage E, from a goat in Australia (GenBank accession number: AF199448), from a dairy cattle in China (GenBank accession number: MN593002), and from a lamb in Ethiopia (GenBank accession number: KT922264). Finally, a mixed A+E infection was detected, based on overlapping peaks at the diagnostic positions, in one Alpine chamois (RR11).

### 2.2. Gdh, Bg and Tpi Phylogenetic Analysis

**Figure 1:**
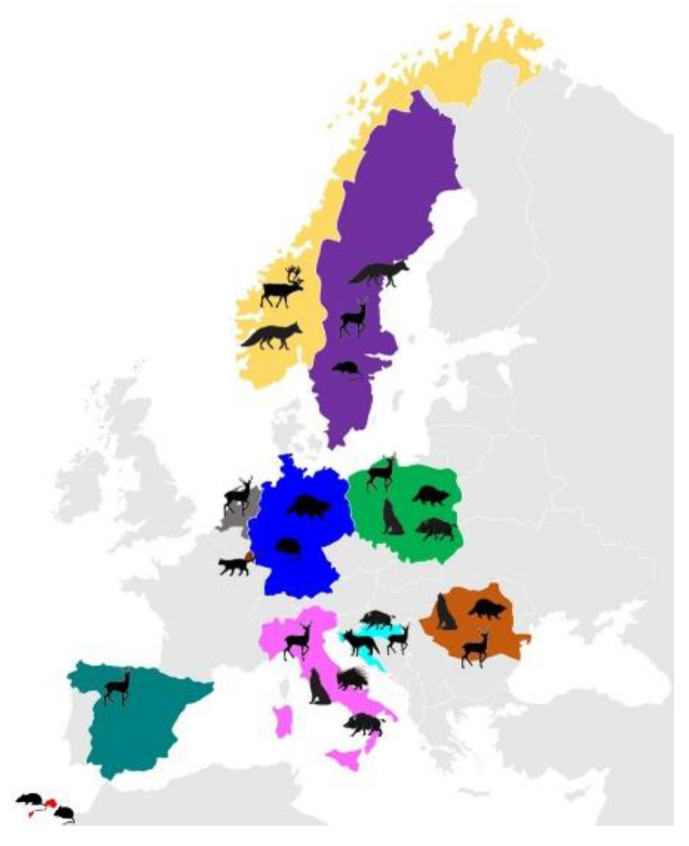
Map showing the geographical provenience of *G. duodenalis* isolates from wild hosts in Europe analyzed in the present study.

Fifteen new sequences from 10 samples were successfully obtained for *gdh*, *tpi*, and *bg* and compared for phylogenetic purposes with previously published data from different wild hosts in Europe (Figure 1).

**Figure 2:**
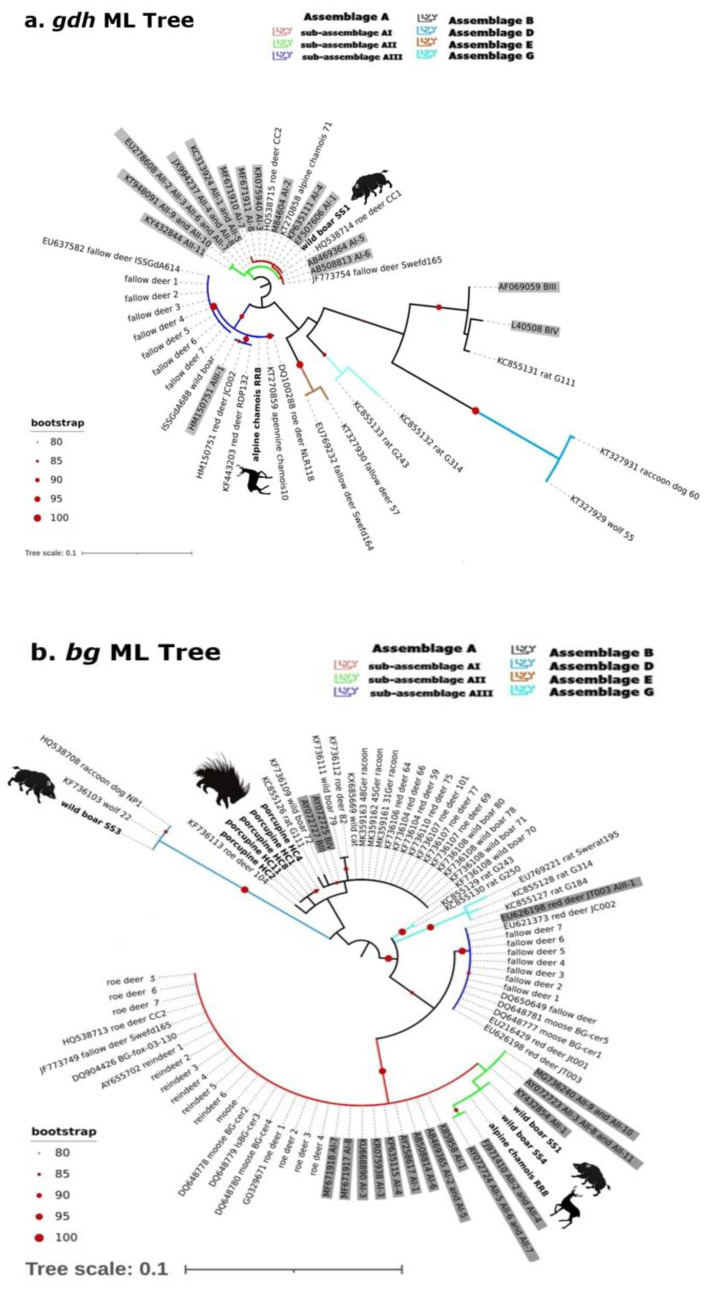

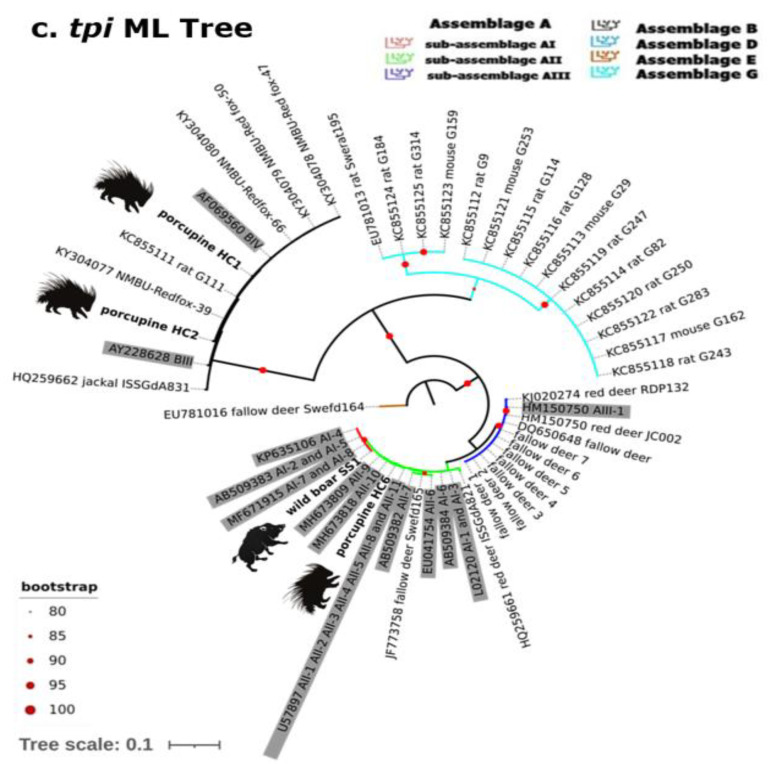
Phylogenetic relationships of *gdh* (**a**), *bg* (**b**) and *tpi* (**c**) sequences of *Giardia* isolates from European wild hosts (Database I–III). The analyses were performed using Maximum Likelihood (ML), with bootstrap values represented as red filled circles (values higher than 80 are shown). Colored clades described the assemblage/sub-assemblage assignments (see Results) and reference sequences are underlined in grey color. Sequences of the present study are in bold.

In the phylogenetic trees achieved using the three genetic markers (see Figure 2a–c), most of the isolates here analyzed belonged to the assemblage A and B, as detailed below.

Molecular analysis of partial *gdh* sequences (Figure 2a; Table 1) revealed well-defined clades describing the different genetic assemblages/sub-assemblages (see Figure 2a). The Clade I, characterized by two sub-clades (green: sub-assemblage AI; red: sub-assemblage AII) included 18 sequences; in particular, our isolate from wild boar (SS1), together with other isolates from ruminants (Italian Alpine chamois, two roe deer from Poland, and one fallow deer from Sweden), was identified as assemblage A-AI. Clade II (blue clade) was well supported (>80 of bootstrap value) and grouped our Alpine chamois (RR8) with 15 isolates from different hosts and countries including Italy, all belonging to assemblage A-AIII. The remaining clades defined the other assemblages B, D, E, and G (all strongly supported, >90 of bootstrap values).

When analyzing the *bg* gene, the wild boar isolate (SS3) was assigned to the host-specific assemblage D (max value of bootstrap), clustering with the corresponding *bg* gene sequence obtained from the *Nyctereutes procyonoides* isolate NP1 (GenBank accession number: HQ538708) and from *Canis lupus* (wolf) isolate 22 (GenBank accession number: KF736103), both detected in Poland (differing by one single-nucleotide polymorphism due to overlapping peaks). The taxonomic assignment of the other isolates was more difficult, due to the low bootstrap values: the two isolates from wild boar (SS1 and SS4) and the isolate from Alpine chamois (RR8) grouped together, being similar but not identical to sequences described as assemblage A-AII (GenBank accession number: AY072723, AY072724), presenting two single-nucleotide polymorphisms (SNPs), in contrast to the results obtained by *gdh* and *tpi* phylogenetic analysis (Figure 2b). Finally, we were able to assign all isolates from porcupines (HC1, HC2, HC4, HC8, and HC11) to the assemblage B (black clade, bootstrap value: 79) together with isolates from different hosts and countries; however, due to the low resolution capability of this marker to discriminate between the sub-assemblages BIII and BIV, it was not possible to identified these isolates at the sub-assemblage level.

Concerning *tpi* analysis, we were able to assign our isolates to well-defined clusters (bootstrap values 70–100, see in detail Figure 2c): in particular, the wild boar SS1 grouped within the assemblage A-AI, as observed in *gdh* phylogenetic analysis, and one porcupine (HC6) was assigned to the assemblage A-AII together with red deer and fallow deer from Croatia and Sweden. The other two *Giardia* porcupine isolates (HC1 and HC2) grouped within the assemblage B-BIV with red foxes and a rat from Sweden and the Canary Island, respectively.

### 2.3. Haplotype Variability

**Figure 3:**
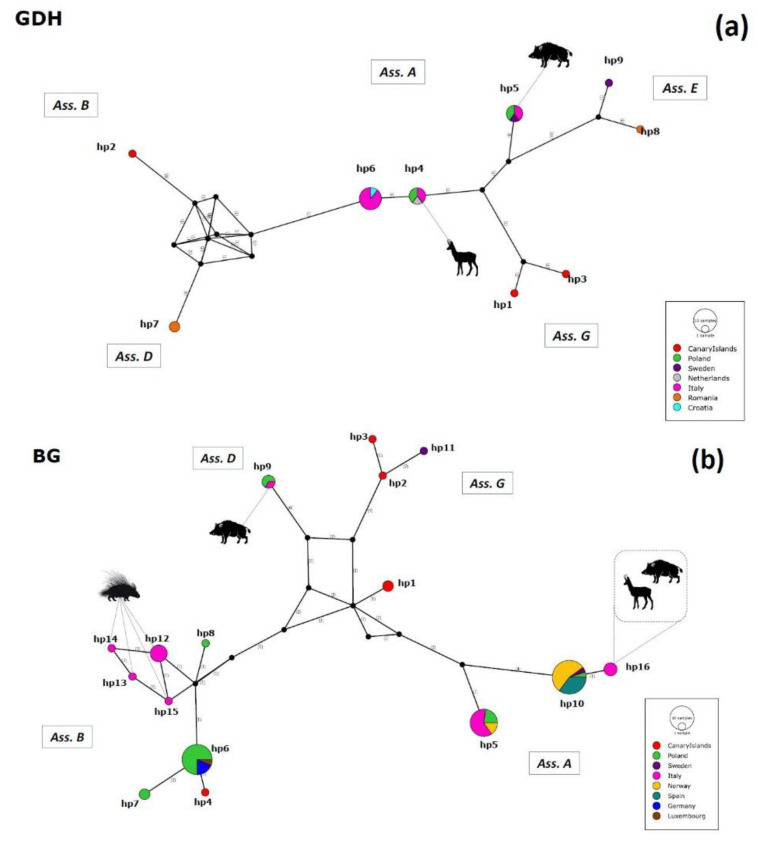

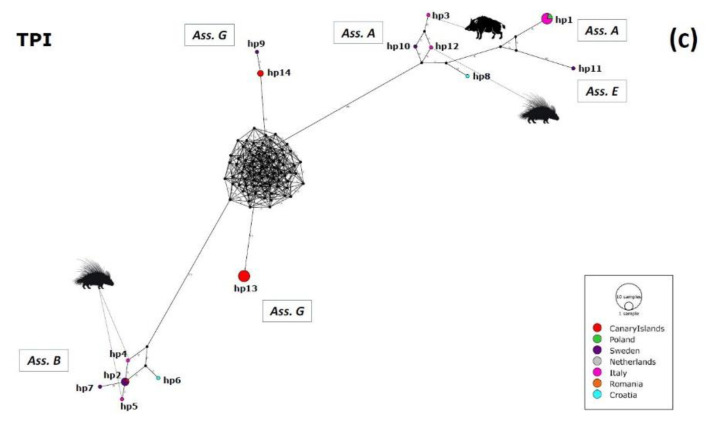
Gdh (**a**), bg (**b**), and tpi (**c**) Median-Joining haplotype networks built in PoPART software. Haplotypes are represented by circles proportional to relative haplotypes abundance; different colors indicate different areas of origin (see Figure 1 for more details). Numbers in brackets refer to the mutational steps between haplotypes. Black circles represent hypothetical missing haplotypes predicted by the model. Hosts from the present study are represented as black silhouettes. Correspondence between different haplotypes and isolates is indicated in Tables S2–S4.

Thirty-nine different haplotypes were identified at the *gdh*, *bg*, and *tpi* loci; in particular, the *bg* locus showed the higher haplotype diversity (Hd). Haplotype variability results are displayed in Tables S1–S4 and visualized as networks representation (Figure 3a–c).

As for the *gdh* locus (Dataset I), nine haplotypes (hp1–hp9) were detected. In particular, haplotype 6 (hp6), recognized within assemblage A, had the highest frequency, followed by haplotype 4 (hp4), just one mutation step from hp6. Hp6 was detected in Italy and Croatia from fallow deer and wild boar, respectively, and corresponds to sub-assemblage AIII.

The Median-Joining Network (Figure 3a) confirmed all the haplotypes retrieved from present study were also shared with others European countries such as Poland, Sweden, and the Netherlands. Indeed, the Alpine chamois isolate from the present study (RR8) clustered together with two red deer isolates from Poland, one Apennine chamois isolate from Italy, and one roe deer isolate from the Netherlands in hp4; while our wild boar isolate SS1 clustered together with two roe deer isolates from Poland, one Apennine chamois isolate from Italy, and one fallow deer isolate from Sweden in haplotype 5 (hp5).

The analysis of Dataset II (*bg*) revealed the presence of 16 different haplotypes (hp1–hp16). Haplotype 10 (hp10) showed the highest frequency, followed by haplotype 6 (hp6). They belong respectively to assemblage A and B. Hp10 was detected in Poland, Sweden, Norway, and Spain in roe deer, fallow deer, fox, reindeer, and moose. Hp6 was detected in Luxembourg, Poland, and Germany in different hosts such as wild cat, wild boar, roe deer, red deer, and raccoon.

The Median-Joining Network (Figure 3b) showed haplotype 5 (hp5), hp6, and hp10 were abundantly shared between different European countries, without evidence of spatial segregation; in addition, haplotypes retrieved from assemblage B had the highest variability.

Our isolates clustered as follows: wild boar SS3 clustered together with a wolf and a raccoon dog from Poland in haplotype 9 (hp9) (assemblage D); wild boar SS1 and SS4 and Alpine chamois RR8 clustered together in haplotype 16 (hp16); haplotype 12 (hp12) included isolates HC1 and HC4 and one variant from the ambiguous isolates HC2, HC8, HC11, all from crested porcupines, which also split with their second variant in haplotype 13 (hp13), haplotype 14 (hp14), and haplotype 15 (hp15).

Finally, the analysis of Database III (*tpi*) showed 14 different haplotypes (hp1–hp14). Haplotype 13 (hp13), belonging to assemblage G, revealed the highest frequency together with haplotype 1 (hp1), from assemblage A. Hp13 was detected in the Canary Islands from rodents (rats and mice); instead, hp1 was recognized in red deer and fallow deer from Poland and Italy, respectively.

The Median-Joining Network (Figure 3c) indicated four well distinct clusters: one including assemblage B, one including assemblages A and E, and two different clusters describing assemblage G, indicating a high internal diversity.

Isolates from the present study clustered individually as different haplotypes, i.e. the wild boar isolate (SS1) in haplotype 3 (hp3) and the isolates from crested porcupines (HC1, HC2, HC6) in haplotype 4 (hp4), haplotype 5 (hp5), and haplotype 12 (hp12), respectively.

## 3. Discussion

There is now strong evidence that parasites produce a significant impact on wildlife population dynamics, acting as evolutionary drivers of traits such as host genetic and phenotypic diversity. However, much of the research is focused on determining the role of wildlife populations as reservoir of parasitic diseases for domestic animals and humans. *G. duodenalis* is a ubiquitous enteric protozoan able to infect a broad range of vertebrates including humans, with different degrees of host specificity depending on the genetic identity of the isolates [9]. In the present study, *G. duodenalis* sequences from different wild animals in Italy were identified and compared with those available from other wild host species in Europe (last update, December 2021).

In Italy, few data are so far available on *G. duodenalis* genotypes diversity in wildlife. The genotyping results obtained in the present study include the first report in Italian wild boar and, along with those previously described by Coppola et al. [21], represent the only data on *G. duodenalis *genotypes in Italian wild rodents.

Sequencing at the *SSU-rRNA* locus indicated that assemblage A was the only detected in wild boar. However, conflicting evidence was found in assemblage/sub-assemblage assignment. As observed by the phylogenetic analysis, the wild boar isolate SS1 was assigned to sub-assemblage AI at both *gdh* and *tpi* loci but to sub-assemblage AII at the *bg* locus, while the isolates SS3 was assigned to the canid-specific assemblages D. Ambiguous results were observed also for isolates from other hosts. Among Alpine chamois, the assemblages A and E, commonly associated with wild hoofed mammals, were found at the *SSU-rRNA* locus, and the sub-assemblage AIII at the *gdh* locus was identified in one isolate (RR8). However, this result was not confirmed at the *bg* locus, which identified the same isolate as sub-assemblage AII, mostly found in humans. The sub-assemblage AIII has been almost exclusively observed in wild ruminants, especially in species within the Family Cervidae [9]. It was described for the first time in fallow deer from northern Italy [18,19] and in Apennine chamois from central Italy [20]. For porcupines, even though sequencing at the *SSU-rRNA* locus assigned all isolates to assemblage B, the detection of assemblage A-AII was obtained for one isolate at the *tpi* locus, while *bg* analysis failed to discriminate other samples between the BIII and BIV sub-assemblages. Lack of concordance is frequently observed in *Giardia* genotyping, mainly due to the presence of genetic differences at a genetic locus (ASH, allelic sequence heterozygosity) within isolates and the frequent occurrence of infections with mixed assemblages [9]. As for carnivores, assemblage D, identified in the present study in wolf from the Apennines of North-Central Italy together with the isolates from wolf from Apennines of South-Central Italy (sequence homology 99% with assemblage C) [33], confirmed the circulation of the canid-specific assemblages C and D in this wild species in Italy.

Several studies reported data on assemblage/sub-assemblage identity of *G. duodenalis* in wild animals in Europe. Assemblage G is predominant in small rodents, mainly rats, as reported from the Canary Island (Spain) and Sweden [22,25], while in Germany, few isolates from mice and voles were found positive for the potentially zoonotic assemblages A and B [17]. In wild hoofed animals from Norway, Poland, Croatia, Romania, Sweden, and The Netherlands, both assemblages A and B were found to a large extent in cervids (e.g., red deer, roe deer, fallow deer, moose, and reindeer) as well as in wild boar [13,14,15,23,26], while the “classical” bovine specific assemblage E was rarely detected in these hosts [24,25]. Canid-specific assemblages C and D have been identified in wolves and raccoon dogs in Croatia, Romania, and Poland [14,15,24,29]. However, among carnivores, also assemblages A and B have been seen in wolves, red foxes, and jackal in Norway, Sweden, and Croatia [14,16,27] and in a free-living European wildcat from Luxembourg [30].

The phylogenetic analysis performed in the present study allowed better depicting the taxonomic position of the Italian isolates, compared with those identified in European wildlife. The genetic variants reported in Italy were found in several countries and, although some clustering relating to the host was observed, all isolates are shared among wildlife of different geographical origins. Differences in local environmental and ecological conditions (e.g., sharing of pastures with domestic animal, possible contacts with humans, access to urban environments) might have a role in the distribution pattern of *G. duodenalis* assemblages in wildlife.

These findings are consistent with results obtained by haplotype analysis. Different haplotypes were shared between diverse wild hosts from several European countries. This variability is mostly represented by assemblage B, confirming its complex genetic diversity [34]. The occurrence of the most represented haplotypes for assemblages A and B, without any evidence of host or spatial segregation, should be considered for a hypothetical analysis of zoonotic transmission risk. 

Our analysis of genotypes diversity of *Giardia* from different hosts and countries allowed taking a step forward for a better knowledge of the geographical distribution and spatial patterns of *G. duodenalis* genotypes in Italian and European wildlife. No novel potentially host-specific genotypes were found in the wildlife surveyed. The results suggest that *Giardia* populations in wild animals are well established and mixed throughout different countries, as previously indicated [35,36]. As an example, to date, the unique *Giardia* sequence available from porcupine worldwide (isolate 10707 from New Zealand, GenBank accession number: KY124019) was identified as assemblage B, along with our isolates from Italy.

However, genetic variation still appears underestimated due to data biases (e.g., low sampling, neglected sequence submission to genetic databases, different amplification targets, low phylogenetic consensus of the current genotyping genes) [9,37]. New genomic approaches are advocated to accurately genotype *G. duodenalis* at the assemblage/sub-assemblage level and to assess its transmission routes reliably.

## 4. Materials and Methods

### 4.1. Sampling

In the period 2014–2019, 23 fecal samples tested positive to the presence of *Giardia* spp. cysts by a direct immunofluorescence assay (Merifluor^®^ Meridian Diagnostic, Cincinnati, OH, USA); they were obtained from 12 crested porcupines (*Hystrix cristata*) collected in the province of Pisa, 4 wild boars (*Sus scrofa*) and 1 wolf (*Canis lupus italicus*) originating from the province of Pistoia (Tuscany, Central, Italy), and 6 Alpine chamois (*Rupicapra rupicapra rupicapra*) harvested in the Italian Central Alps. All samples were subjected to molecular and phylogenetic analyses for assemblage/sub-assemblage identification.

### 4.2. Assemblage and Sub-Assemblage Identification

Genomic DNA was isolated using the QIAmp DNA stool mini kit (QIAGEN, Valencia, CA, USA) following the manufacturer’s instructions. All samples were amplified by two-step nested PCR protocols using four sets of primers targeting different loci, according to previous descriptions: (i) RH4 and RH11 for primary PCR and GiarR and GiarF for secondary PCR [38] designed to amplify a 130 bp fragment of the small subunit 18S (*SSU rRNA*) gene; (ii) external forward primer GDHeF, internal forward primer GDHiF, and reverse primer GDHiR designed to yield a fragment of 432 bp of the glutamate dehydrogenase (*gdh*) gene [39]; (iii) forward primer G7, forward inner primer G376, and reverse primer G759 to amplify a 384 bp fragment of the β-giardin (bg) locus [40]; (iv) primers AL3543 and AL3546 for primary PCR and AL3544 and AL3545 for secondary PCR which amplified a fragment of 530 bp of the triose phosphate isomerase (*tpi*) gene [41].

Amplicons were purified using a mi-PCR Purification Kit (Metabion International AG) and sent to an external laboratory for sequencing (Bio-Fab Research, Rome, Italy). The resulting chromatograms were manually checked using Finch TV 1.4 software (Geospiza, Inc., Seattle, WA, USA), in order to identify possible double peaks for mixed infections or single-nucleotide polymorphisms (SNPs). After adjoining of the forward and reverse reads, the sequences included approximately 60% of positions readable on both strands. The sequences were pairwise aligned using Clustal Omega software [42] to recheck variable positions. Consensus sequences for each amplified region were compared to those previously published in GenBank database: particularly, *18S SSU rRNA* identities at assemblage level were verified using the Basic Local Alignment Search Tool (BLAST). Subsequently, sequences were trimmed to the shortest length with high quality in all our samples and complete representation in the downloaded ones.

GiardiaDB (https://giardiadb.org/), a central resource for public access to computational platforms, analysis tools, and data mining of genome-scale research data, was used to build three datasets (I, II, and III), one for each genetic marker (*gdh*, *bg*, and *tpi*) analyzed for phylogenetic purposes. In detail, we used a multistep strategy (section: GiardiaDB’s My Strategies) matching the keywords “*Giardia* sp./*Giardia*” and “*Giardia duodenalis*” together with “Europe”, excluding all the sequences isolated from humans (*Homo sapiens*). This produced a unique database with more than 100 sequences that was trimmed down by removing all sequences not isolated from wild animals and/or characterized in different genetic loci (*ITS*, *SSU rRNA*). In addition, a final GenBank “complete record” check was performed (last update December 2021) for each sequence, paying attention to “Features—isolation and host”.

Three datasets were thus obtained, defined as [i] Dataset I: including 26 *gdh* sequences (plus 16 as assemblage/sub-assemblage reference sequences, accession numbers: KY432844, KT948091, EU278608, JX994237, KC313924, MF671910-11, KR075940, M84604, KP635111, EF507606, AB469364, AB508813, HM150751 for assemblage A; AF069059, L40508 for assemblage B); [ii] Dataset II: including 69 *bg* sequences (plus 17 as Assemblage/sub-assemblage reference sequences, accession numbers: EU626198, X85958, AB469365, AB508814, AY258617, KP635115, KP075938, KU668890, MF671917-18, FJ971410, AY072723-4, MG736240, KY432854 for assemblage A; AY072725, AY072727 for assemblage B); and [iii] Dataset III; characterized by 38 *tpi* sequences (plus 13 as assemblage/sub-assemblage reference sequences, accession numbers: HM150750, L02120, U57897, AB509382-84, KP635106, MF671915, MH673809, MH673818, EU041754 for assemblage A; AF069560, AY228628 for assemblage B), and were aligned by MUSCLE v3.8 [43]. The assemblage/sub-assemblage A reference sequences were selected from Cai et al. [44].

After alignment, each dataset was trimmed at particular nucleotide positions of the reference sequences, to maximize the overlap between ours and *Giardia* sequences from European wild hosts (Table 1); this produced valid alignments for 42 *gdh* sequences of 433 bp (trimmed at positions 461 to 894 of the reference M84604), 86 *bg* sequences of 180 bp (trimmed at positions 1034 to 1214 of the reference X85958), and 51 *tpi* sequences of 530 bp (trimmed at positions 499 to 1029 of the reference L02120).

The best-fit model of evolution for each alignment was determined using the Akaike Information Criteria (AIC) selected from Modeltest v3.7 [45], included in the “phangorn package” in Rstudio v1.4.1106. Phylogenetic analyses of Datasets I, II, and III were conducted using the Maximum Likelihood (ML) statistical method and 1000 bootstrap pseudo-replications using Rstudio v1.4.110 (“ape” and “phangorn” packages) under the TrN+G+I substitution model (Dataset I), TrN+I (Dataset II), and TIM1+I (Dataset III). For the details of the datasets, see Table 1. Phylogenetic Trees were visualized trough Itol: Interactive Tree of life v6 (https://itol.embl.de/). The sequences generated in this study were deposited in GenBank under accession numbers OL840340-OL840345 for the *SSU rDNA* gene, OL828750-OL828751 for the *gdh* locus, OL944442-OL944447 for the *bg* gene, and OL944434-OL944437 for the *tpi* locus.

### 4.3. Haplotype Analysis and Networks

To gain insights into the geographical distribution of assemblages/sub-assemblages in the European context of wildlife, a haplotype analysis was conducted on polymorphic sites using DnaSP v.6 software [46] and Tajima’s D test [47]. PoPART (Population Analysis with Reticulate Trees) genetic software [48] was used to perform the Median-Joining network calculation [49]. The analysis was performed on the three databases (see Section 4.2 of Materials and Methods and Table 1) with sequences trimmed to the shortest length with high-quality fragments and sites, without considering alignment gaps; in detail, *gdh* (Dataset I), consisting of 26 sequences of 154 bp, *bg* (Dataset II), of 69 sequences of 155 bp, and *tpi* (Dataset III), of 38 sequences of 433 bp, including also ambiguous sequences (double peaks presence). In case of ambiguous sequences (unphased data), haplotype reconstruction was performed in DnaSP v.6 by the algorithms provided by PHASE [50,51] which uses a coalescent-based Bayesian method to infer haplotypes.

## Figures and Tables

**Table 1:** Materials analyzed in this study, with details on the host species, classification, and country of isolation as reported in GenBank or in references. Sequences retrieved from GenBank (Dataset I–III) were used for phylogenetic and network analyses. The symbol “ indicates that information is as above in the same column.

Host Species	Origin	Specimen Code	Assemblage/ Sub-Assemblage (n)	GenBank Accession Number	Ref.
***SSU_rRNA***					
*Rupicapra r. rupicapra*	“	RR6; RR7; RR8; RR9; RR11; RR12	A(3); E(2); A+E(1)	OL840340-42	present study
*Canis lupus*	“	CL1	D	OL840343	present study
*Sus scrofa*	“	SS1-SS4	A(4)	OL840344	present study
*Hystrix cristata*	“	HC1-HC12	B(12)	OL840345	present study
**Dataset I *gdh***					
*Rupicapra r. rupicapra*	“	RR8	AIII	OL828750	present study
*Sus scrofa*	“	SS1	AI	OL828751	present study
*Rattus rattus*	Spain: Canary Islands	G111; G314; G243	B(1); G(2)	KC855131-33	Fernández-Álvarez et al., 2014 [22]
*Cervus elpahus*	Poland	JC002	A	HM150751	Solarczyk et al., 2012 [23]
*Cervus elpahus*	“	RDP132	A	KF443203	unpublished
*Capreolus capreolus*	“	CC1; CC2	A(2)	HQ538714-15	Solarczyk et al., 2012 [23]
*Sus scrofa*	Croatia	ISSGdA688	A	sequence not deposited ^1^	Beck et al., 2011 [14]
*Canis lupus*	Romania	wolf55	D	KT327929	Adriana et al. 2016 [24]
*Dama dama*	“	deer57	E	KT327930	“
*Nyctereutes procyonoides*	“	racoondog60	D	KT327931	“
*Dama dama*	Sweden	Swefd164; Sewfd165	E(1); A(1)	EU769232; JF773754	Lebbad et al., 2010 [25]
*Capreolus capreolus*	The Netherlands	NLR118	A	DQ100288	van der Giessen et al., 2006 [26]
*Dama dama*	Italy	ISSGdA614;(fallow deer 1–7)	AIII(8)	EU637582	Cacciò et al., 2008 [19]
*Rupicapra r. rupicapra*	“	71	AI	KT270858	De Liberato et al., 2015 [20]
*Rupicapra p. ornata*	“	10	AIII	KT270859	“
**Dataset II *bg***					
*Sus scrofa*	Italy	SS1; SS4; SS3	AII(2) D(1)	OL944445-46	present study
*Hystrix cristata*	“	HC1; HC2; HC4; HC8; HC11	B(5)	OL944442-44	present study
*Rupicapra r. rupicapra*	“	RR8	AII	OL944447	present study
*Rattus rattus*	Spain: Canary Island	G111; G184; G314; G243; G250	B(1); G(4)	KC855126-30	Fernández-Álvarez et al., 2014 [22]
*Cervus elaphus*	Poland	JC002	A	EU621373;	Solarczyk et al., 2012 [23]
*Capreolus capreolus*	“	CC2	A	HQ538713	Solarczyk et al., 2012 [23]
*Cervus elaphus*	Poland	JT001, JT003	A(2)	EU216429; EU626198	unpublished
*Nyctereutes procyonoides*	“	NP1	D	HQ538708	unpublished
*Sus scrofa*	“	70; 71; 78; 80; 72; 79	B(6)	KF736108-09; KF736111	Stojecki et al., 2015 [15]
*Capreolus capreolus*	“	69; 77; 101; 82; 104	B(5)	KF736107; KF736112-13	“
*Canis lupus*	“	22	D	KF736103	“
*Cervus elaphus*	“	59, 66; 64; 75	B(4)	KF736104; KF736106; KF736110	“
*Rattus norvergicus*	Sweden	Swerat195	G	EU769221	Lebbad et al., 2010 [25]
*Dama dama*	“	Swefd165	A	JF773749	“
*Vulpes vulpes*	Norway	BG-fox-03-130	A	DQ904426	Hamnes et al., 2007 [27]
*Rangifer tarandus*	“	(reindeer 1–6)	A(6)	sequence not deposited ^2^	Robertson et al., 2007 [13]
*Alces alces*	“	(moose)	A	sequence not deposited ^2^	“
*Alces alces*	“	BG-cer1; BG-cer2; BG-cer3; BG-cer4;BG-cer5	A(5)	DQ648777-81	“
*Capreolus capreolus*	Spain	(roe deer 1–7)	AII(7)	sequence not deposited ^3^	García-Presedo et al., 2013 [28]
*Dama dama*	Italy	(fallow deer 1–8)	AIII(8)	DQ650649	Lalle et al., 2007 [18]
*Procyon lotor*	Germany	31; 45; 48	BIV(3)	MK359161-63	Solarczyk et al., 2021 [29]
*Felis silvestris*	Luxembourg	FS1	B	KX685669	Solarczyk et al., 2019 [30]
**Dataset III *tpi***					
*Sus scrofa*	Italy	SS1	AI	OL944434	present study
*Hystrix cristata*	“	HC1; HC2; HC6;	BIV(2); AII(1)	OL944435-37	present study
*Rattus rattus*	Spain: Canary Island	G111; G9; G82; G114; G128; G243; G247; G250; G283; G184; G314	B(1); G(10)	KC855111-12; KC855114-16; KC855118-20; KC855122; KC855124-25	Fernández-Álvarez et al., 2014 [22]
*Mus musuculus domesticus*	“	G29; G162; G253; G159	G(4)	KC855113; KC855117; KC855121; KC855123	“
*Cervus elaphus*	Poland	JC002	A	HM150750	Solarczyk et al., 2012 [23]
*Cervus elaphus*	“	RDP132	A	KJ020274	unpublished
*Cervus elaphus*	Croatia	ISSGdA821	A	HQ259661	Beck et al., 2011 [14]
*Canis aureus moreoticus*	“	ISSGdA831	B	HQ259662	“
*Rattus norvegicus*	Sweden	Swerat195	G	EU781013	Lebbad et al., 2010 [25]
*Dama dama*	“	Swefd164; Swefd165	E(1); A(1)	EU781016; JF773758	“
*Vulpes vulpes*	“	NMBU-Red fox-39; NMBU-Red fox-47; NMBU-Red fox-50; NMBU-Red fox-66	B(4)	KY304077-80	Debenham et al., 2017 [16]
*Dama dama*	Italy	(fallow deer 1–8)	AIII(8)	DQ650648	Lalle et al., 2007 [18]

^1^ Sequence identical to EU637582 (Cacciò et al., 2008) [19]; ^2^ Sequence identical to AY655702 (Trout et al., 2004) [31]; ^3^ Sequence identical to GQ329671 (Lebbad et al., 2011) [32].

## Supplementary Materials

The following supporting information can be downloaded from https://www.mdpi.com/article/10.3390/pathogens11010105/s1, Table S1: Haplotype variability; Table S2: *Gdh* locus haplotype analysis, Table S3: *Bg* locus haplotype analysis, and Table S4: *Tpi* locus haplotype analysis.

## References

[1] Hudson P.J., Rizzoli A.P., Grenfell B.T., Heesterbeek H., Dobson A.P. (2002). The Ecology of Wildlife Diseases.

[2] Gómez A., Nichols E. (2013). Neglected wild life: Parasitic biodiversity as a conservation target. Int. J. Parasitol. Parasites Wildl..

[3] Stringer A.P., Linklater W. (2014). Everything in Moderation: Principles of Parasite Control for Wildlife Conservation. BioScience.

[4] Hudson P.J., Dobson A.P., Lafferty K.D. (2006). Is a healthy ecosystem one that is rich in parasites?. Trends Ecol. Evol..

[5] Lafferty K.D., Allesina S., Arim M., Briggs C.J., De Leo G., Dobson A.P., Dunne J.A., Johnson P.T., Kuris A.M., Marcogliese D.J. (2008). Parasites in food webs: The ultimate missing links. Ecol. Lett..

[6] De Castro F., Bolker B. (2005). Mechanisms of disease-induced extinction. Ecol. Lett..

[7] Heard M.J., Smith K.F., Ripp K.J., Berger M., Chen J., Dittmeier J., Goter M., McGarvey S.T., Ryan E. (2013). The threat of disease increases as species move toward extinction. Conserv. Biol..

[8] Adam R.D. (2021). Giardia duodenalis: Biology and Pathogenesis. Clin. Microbiol. Rev..

[9] Feng Y., Xiao L. (2011). Zoonotic potential and molecular epidemiology of Giardia species and giardiasis. Clin. Microbiol. Rev..

[10] Cacciò S.M., Lalle M., Svärd S.G. (2018). Host specificity in the Giardia duodenalis species complex. Infect. Genet. Evol..

[11] Seabolt M.H., Konstantinidis K.T., Roellig D.M. (2021). Hidden Diversity within Common Protozoan Parasites as Revealed by a Novel Genotyping Scheme. Appl. Environ. Microbiol..

[12] Hulme P.E. (2014). Invasive species challenge the global response to emerging diseases. Trends Parasitol..

[13] Robertson L.J., Forberg T., Hermansen L., Hamnes I.S., Gjerde B. (2007). Giardia duodenalis cysts isolated from wild moose and reindeer in Norway: Genetic characterization by PCR-rflp and sequence analysis at two genes. J. Wildl. Dis..

[14] Beck R., Sprong H., Lucinger S., Pozio E., Caccio S.M. (2011). A large survey of Croatian wild mammals for Giardia duodenalis reveals a low prevalence and limited zoonotic potential. Vector Borne Zoonotic Dis..

[15] Stojecki K., Sroka J., Cacciò S.M., Cencek T., Dutkiewicz J., Kusyk P. (2015). Prevalence and molecular typing of Giardia duodenalis in wildlife from eastern Poland. Folia Parasitol..

[16] Debenham J.J., Landuyt H., Troell K., Tysnes K., Robertson L.J. (2017). Occurrence of Giardia in Swedish Red Foxes (Vulpes vulpes). J. Wildl. Dis..

[17] Helmy Y.A., Spierling N.G., Schmidt S., Rosenfeld U.M., Reil D., Imholt C., Jacob J., Ulrich R.G., Aebischer T., Klotz C. (2018). Occurrence and distribution of Giardia species in wild rodents in Germany. Parasit. Vectors.

[18] Lalle M., Frangipane di Regalbono A., Poppi L., Nobili G., Tonanzi D., Pozio E., Cacciò S.M. (2007). A novel Giardia duodenalis assemblage A subtype in fallow deer. J. Parasitol..

[19] Cacciò S.M., Beck R., Lalle M., Marinculic A., Pozio E. (2008). Multilocus genotyping of Giardia duodenalis reveals striking differences between assemblages A and B. Int. J. Parasitol..

[20] De Liberato C., Berrilli F., Marangi M., Santoro M., Trogu T., Putignani L., Lanfranchi P., Ferretti F., D’Amelio S., Giangaspero A. (2015). Giardia duodenalis in Alpine (Rupicapra rupicapra rupicapra) and Apennine (Rupicapra pyrenaica ornata) chamois. Parasit. Vectors.

[21] Coppola F., Maestrini M., Berrilli F., Guadano Procesi I., Felicioli A., Perrucci S. (2020). First report of Giardia duodenalis infection in the crested porcupine (Hystrix cristata L., 1758). Int. J. Parasitol. Parasites Wildl..

[22] Fernández-Álvarez Á., Martín-Alonso A., Abreu-Acosta N., Feliu C., Hugot J.P., Valladares B., Foronda P. (2014). Identification of a novel assemblage G subgenotype and a zoonotic assemblage B in rodent isolates of Giardia duodenalis in the Canary Islands, Spain. Parasitology.

[23] Solarczyk P., Majewska A.C., Moskwa B., Cabaj W., Dabert M., Nowosad P. (2012). Multilocus genotyping of Giardia duodenalis isolates from red deer (Cervus elaphus) and roe deer (Capreolus capreolus) from Poland. Folia Parasitol..

[24] Adriana G., Zsuzsa K., Mirabela Oana D., Mircea G.C., Viorica M. (2016). Giardia duodenalis genotypes in domestic and wild animals from Romania identified by PCR-RFLP targeting the gdh gene. Vet. Parasitol..

[25] Lebbad M., Mattsson J.G., Christensson B., Ljungström B., Backhans A., Andersson J.O., Svärd S.G. (2010). From mouse to moose: Multilocus genotyping of Giardia isolates from various animal species. Vet. Parasitol..

[26] Van der Giessen J.W.B., De Vries A., Roos M., Wielinga P., Kortbeek L.M., Mank T.G. (2006). Genotyping of Giardia in Dutch patients and animals: A phylogenetic analysis of human and animal isolates. Int. J. Parasitol..

[27] Hamnes I.S., Gjerde B.K., Forberg T., Robertson L.J. (2007). Occurrence of Giardia and Cryptosporidium in Norwegian red foxes (Vulpes vulpes). Vet. Parasitol..

[28] García-Presedo I., Pedraza-Díaz S., González-Warleta M., Mezo M., Gómez-Bautista M., Ortega-Mora L.M., Castro-Hermida J.A. (2013). The first report of Cryptosporidium bovis, C. ryanae and Giardia duodenalis sub-assemblage A-II in roe deer (Capreolus capreolus) in Spain. Vet. Parasitol..

[29] Solarczyk P., Dabert M., Frantz A.C., Osten-Sacken N., Trzebny A., Wojtkowiak-Giera A., Heddergott M. (2021). Zoonotic Giardia duodenalis sub-assemblage BIV in wild raccoons (Procyon lotor) from Germany and Luxembourg. Zoonoses Public Health.

[30] Solarczyk P., Osten-Sacken N., Frantz A.C., Schneider S., Pir J.B., Heddergott M. (2019). First molecular detection of Giardia duodenalis assemblage B in a free-living European wildcat (Felis s. silvestris) from Luxembourg. Acta Protozool..

[31] Trout J.M., Santín M., Greiner E., Fayer R. (2004). Prevalence of Giardia duodenalis genotypes in pre-weaned dairy calves. Vet. Parasitol..

[32] Lebbad M., Petersson I., Karlsson L., Botero-Kleiven S., Andersson J.O., Svenungsson B., Svärd S.G. (2011). Multilocus genotyping of human Giardia isolates suggests limited zoonotic transmission and association between assemblage B and flatulence in children. PLoS Negl. Trop. Dis..

[33] Di Francesco C.E., Smoglica C., Paoletti B., Angelucci S., Innocenti M., Antonucci A., Di Domenico G., Marsilio F. (2019). Detection of selected pathogens in Apennine wolf (Canis lupus italicus) by a non-invasive GPS-based telemetry sampling of two packs from Majella National Park, Italy. Eur. J. Wildl. Res..

[34] Mizuno T., Matey E.J., Bi X., Songok E.M., Ichimura H., Tokoro M. (2020). Extremely diversified haplotypes observed among assemblage B population of Giardia intestinalis in Kenya. Parasitol. Int..

[35] Garcia-R J.C., French N., Pita A., Velathanthiri N., Shrestha R., Hayman D. (2017). Local and global genetic diversity of protozoan parasites: Spatial distribution of Cryptosporidium and Giardia genotypes. PLoS Negl. Trop. Dis..

[36] Spotin A., Karamat M., Mahami-Oskouei M., Shahbazi A., Ahmadpour E., Galeh T.M., Fallahi S. (2018). Genetic variability and transcontinental sharing of Giardia duodenalis infrapopulations determined by glutamate dehydrogenase gene. Acta Trop..

[37] Capewell P., Krumrie S., Katzer F., Alexander C.L., Weir W. (2021). Molecular epidemiology of Giardia infections in the Genomic Era. Trends Parasitol..

[38] Read C., Walters J., Robertson I.D., Thompson R.C. (2002). Correlation between genotype of Giardia duodenalis and diarrhoea. Int. J. Parasitol..

[39] Read C.M., Monis P.T., Thompson R.C. (2004). Discrimination of all genotypes of Giardia duodenalis at the glutamate dehydrogenase locus using PCR-RFLP. Infect. Genet. Evol..

[40] Cacciò S.M., De Giacomo M., Pozio E. (2002). Sequence analysis of the beta-giardin gene and development of a polymerase chain reaction-restriction fragment length polymorphism assay to genotype Giardia duodenalis cysts from human faecal samples. Int. J. Parasitol..

[41] Sulaiman I.M., Fayer R., Bern C., Gilman R.H., Trout J.M., Schantz P.M., Das P., Lal A.A., Xiao L. (2003). Triosephosphate isomerase gene characterization and potential zoonotic transmission of Giardia duodenalis. Emerg. Infect. Dis..

[42] Chenna R., Sugawara H., Koike T., Lopez R., Gibson T.J., Higgins D.G., Thompson J.D. (2003). Multiple sequence alignment with the Clustal series of programs. Nucleic Acids Res..

[43] Edgar R.C. (2004). MUSCLE: A multiple sequence alignment method with reduced time and space complexity. BMC Bioinform..

[44] Cai W., Ryan U., Xiao L., Feng Y. (2021). Zoonotic giardiasis: An update. Parasitol. Res..

[45] Posada D. (2008). jModelTest: Phylogenetic model averaging. Mol. Biol. Evol..

[46] Rozas J., Ferrer-Mata A., Sánchez-DelBarrio J.C., Guirao-Rico S., Librado P., Ramos-Onsins S.E., Sánchez-Gracia A. (2017). DnaSP 6: DNA Sequence Polymorphism Analysis of Large Data Sets. Mol. Biol. Evol..

[47] Tajima F. (1989). Statistical method for testing the neutral mutation hypothesis by DNA polymorphism. Genetics.

[48] Leigh J.W., Bryant D. (2015). POPART: Full-feature software for haplotype network construction. Methods Ecol. Evol..

[49] Bandelt H.J., Forster P., Röhl A. (1999). Median-joining networks for inferring intraspecific phylogenies. Mol. Biol. Evol..

[50] Stephens M., Smith N.J., Donnelly P. (2001). A new statistical method for haplotype reconstruction from population data. Am. J. Hum. Genet..

[51] Stephens M., Donnelly P. (2003). A comparison of bayesian methods for haplotype reconstruction from population genotype data. Am. J. Hum. Genet..

