# Supplementary material for: Giardia duodenalis in Wildlife: Exploring Genotype Diversity in Italy and across Europe
Source: Pathogens. 2022 Jan 16;11(1):105. doi: 10.3390/pathogens11010105 (PMC8777849; doi:10.3390/pathogens11010105)
Supplement: Supplementary file 1 [file pathogens-11-00105-s001.zip › pathogens-1539602-supplementary.pdf]

Table S1. Haplotype analysis, polymorphic sites analysis and Tajima's D test estimation performed by DnaSP v.6 software for *gdh*, *bg* and *tpi* loci. Π indicates the nucleotide diversity (average number of nucleotide substitutions between all unique pairwise comparisons of sequences); S indicates the number of segregating sites. The asterisks show significance of Tajima's D test values, where * is equal to not significant (P>0.10 or 0.10 > P > 0.05).

**Gene**	**N. of haplotypes**	**Hd (Haplotype diversity)**	**π (Nucleotide diversity)**	**S (N. segregating sites)**	**Tajima's D**
gdh	9	0.8246	0.05205	33	-0.56486*
bg	16	0.8234	0.04675	29	0.72639*
tpi	14	0.8312	0.12852	145	1.98090 *

Table S2. *Gdh* locus haplotype analysis; * indicates the presence of different haplotypes generated from a single ambiguous (double peaks) sequence retrieved from a single isolate; ** indicates present study isolates.

HAPLOTYPE	N. OF SEQUENCES	HOST (N)	ASSEMBLAGE DETECTED	AREA
hp1	1	*R. rattus* (1)	G	Canary Islands
hp2	1	*R. rattus* (1)	B	Canary Islands
hp3	1	*R. rattus* (1)	G	Canary Islands
hp4	5	*C. elaphus* (2);<br>*R. r. rupicapra* (1)**;<br>*R .p. ornata* (1);<br>*C. capreolus* (1)	A	Poland, Italy,<br>The Netherlands
hp5	5	*C. capreolus* (2);<br>*R. r. rupicapra* (1);<br>*S. scrofa* (1)**; *D. dama* (1)	A	Poland, Italy,<br>Sweden
hp6	9	*D. dama* (8); *S. scrofa* (1)	A	Italy, Croatia
hp7	2	*C. lupus* (1);<br>*N. procyonoides* (1)	D	Romania
hp8	1	*D. dama* (1)	E	Romania
hp9	1	*D. dama* (1)	E	Sweden

Table S3. *Bg* locus haplotype analysis; * indicates the presence of different haplotypes generated from a single ambiguous (double peaks) sequence retrieved from a single isolate; ** indicates present study isolates.

HAPLOTYPE	N. OF SEQUENCES	HOST (N)	ASSEMBLAGE DETECTED	AREA
hp1	2	*R. rattus* (2)	G	Canary Islands
hp2	1	*R. rattus* (1)	G	Canary Islands
hp3	1	*R. rattus* (1)	G	Canary Islands
hp4	1	*R. rattus* (1)	B	Canary Islands
hp5	13	C. *elaphus* (3); *A. alces* (2); *D. dama* (8)	A	Poland, Norway, Italy
hp6	16	*F. silvestris* (1); *S. scrofa* (5); *C. capreolus* (3); *C. elaphus* (4); *P. lotor* (3)	B	Luxembourg, Poland, Germany
hp7	2	*S. scrofa* (1); *C. capreolus* (1)	B	Poland
hp8	1	*C. capreolus* (1);	B	Poland
hp9	3	*C. lupus* (1*); N. procyonoides* (1); *S. scrofa* (1)**	D	Poland, Italy
hp10	20	*C. capreolus* (8); *D. dama* (1); *V. vulpes* (1); *R. tarandus* (6); *A. alces* (4)	A	Poland, Sweden, Norway, Spain
hp11	1	*R. norvegicus* (1)	G	Sweden
hp12	5*	*H. cristata* (5)*,**	B	Italy
hp13	1*	*H. cristata* (1)*,**	B	Italy
hp14	1*	*H. cristata* (1)*,**	B	Italy
hp15	1*	*H. cristata* (1)*,**	B	Italy
hp16	3	*S. scrofa* (2)**; *R. r. rupicapra* (1)**	A	Italy

Table S4. *Tpi* locus haplotype analysis; * indicates the presence of different haplotypes generated from a single ambiguous (double peaks) sequence retrieved from a single isolate; ** indicates present study isolates.

HAPLOTYPE	N. OF SEQUENCES	HOST(N)	ASSEMBLAGE DETECTED	AREA
hp1	10	*C. elaphus* (2); *D. dama* (8)	A	Poland, Italy
hp2	5*	*R. rattus* (1); *V. vulpes* (4)*	B	Canary Islands, Sweden
hp3	1	*S. scrofa* (1)**	A	Italy
hp4	1	*H. cristata* (1)**	B	Italy
hp5	1	*H. cristata* (1)**	B	Italy
hp6	1	*C. a. moreoticus* (1)	B	Croatia
hp7	1*	*V. vulpes* (1)*	B	Sweden
hp8	1	*C. elaphus* (1)	A	Croatia
hp9	1	*R. norvegicus* (1)	G	Sweden
hp10	1	*D. dama* (1)	A	Sweden
hp11	1	*D. dama* (1)	E	Sweden
hp12	1	*H. cristata* (1)**	A	Italy
hp13	11	*R. rattus* (8); *M. musculus* (3)	G	Canary Islands
hp14	3	*R. rattus* (2); *M. musculus* (1)	G	Canary Islands
